# Comparing aggregated and nonlinear measures of coordinative behaviors in immediate responder first aid groups: a controlled experimental simulation study

**DOI:** 10.1186/s41077-026-00501-3

**Published:** 2026-11-02

**Authors:** Wilhelm Brodin, Fredrik Fernlund, Carl-Oscar Jonson, Erik Prytz

**Affiliations:** 1 Department of Computer and Information Science, Linköping University, Linköping, Sweden; 2 Department of Biomedical and Clinical Sciences, Linköping University, Linköping, Sweden; 3 Center for Disaster Medicine and Traumatology, Region Östergötland, Linköping, Sweden

**Keywords:** Nonlinear dynamics, Multidimensional recurrence quantification analysis, Ad-hoc teams, Co-ACT, Teamwork, First aid, Accidental injuries, Cardiopulmonary resuscitation, Simulator

## Abstract

**Background:**

When accidents and disasters happen, emergent ad-hoc groups of immediate responders form to provide aid before the arrival of professional medical responders. Little is known about how immediate responders coordinate with each other over time and how that coordination affects the quality of the first aid that is provided. Emergency response is described as complex and chaotic, but methods for analyzing complexity have rarely been applied in the emergency response context. This exploratory study aims to compare recurrence quantification analysis and aggregated data analysis of coordination behaviors by immediate responders to predict performance in groups providing first aid in a simulated context.

**Methods:**

Sixteen groups of three participants (*N* = 48, 58% male, 40% female, and 2% preferred not to answer) were recruited from a large university and the surrounding city in southern Sweden using convenience sampling. They took part in a simulated traffic accident scenario. Of the participants, 56% had prior first aid training, including 6 emergency response professionals who were individually placed in different groups. The first aid performance of each group was rated by three subject matter experts. Only the first 10 min and 28s of each scenario was included in the analysis to allow for direct comparison of the recurrence quantification analysis results between groups. The Co-ACT framework was used to code participants’ coordinative behaviors, and the dynamics of the behaviors’ structure were calculated using a multidimensional recurrence quantification analysis. The aggregated and dynamical structures of the coordinative behaviors (i.e. behaviors of team members coordinating teamwork) were evaluated for their predictive power with regard to first aid performance ratings.

**Results:**

Two groups were excluded due to incomplete data, and the analyses were conducted on the remaining 14 groups. Rate of recurrence of coordinative behaviors was found to significantly predict first aid performance (*p =* .02, *β = -0.57*) while aggregated post-experimental measures of coordinative behaviors did not (Action implicit *p* = .37, *β = -0.31*, Action explicit *p* = .97, *β = <0.01*, Information implicit *p* = .99, *β = 0.01*, Information explicit *p* = .68, *β = -0.14*). The negative effect of recurrence rate of coordinative behaviors on first aid performance suggests that other behaviors not measured in the Co-ACT analysis (e.g., task work such as chest compressions or bleeding control) had more effect on the quality of the first aid that was provided, at least in the current scenario.

**Conclusions:**

The study shows the usefulness of controlled scenarios for studying complexity in provision of first aid. Additionally, the results provide initial evidence for the predictive power of recurrence rate on first aid performance in ad-hoc first aid groups. This exploratory study thus shows initial evidence of the usefulness of complexity methods to understand the emergency response context. These contributions have potential significance for both emergency response researchers and practitioners.

## Introduction

When accidents and disasters happen, people who are affected or simply nearby will typically mount an immediate aid response before the arrival of professional emergency responders. These immediate responders can profoundly impact the success of the response to accidents and disasters, as well as recovery after the fact [1]. When immediate responders perceive an unmet need in an emergency and expect that the professional response will be insufficient, they will spontaneously form groups with a shared goal of filling the unmet needs [2]. As these groups are constituted of individuals who are affected by the emergency or who happened to be close by, the level of knowledge, familiarity, and trust present in the group can vary greatly. There is a need for these emergent groups to work together, based on the expertise present in the group, to function properly and provide effective aid by coordinating both actions and information [3].

Teamwork consists of explicit or implicit coordination of actions or information [4]. Explicit coordination of actions or information is direct and unambiguous. By contrast, implicit coordinative behavior is more anticipatory and tacit. A review of coordination by in-hospital resuscitation teams found that information coordination in the form of planning and leadership communication improved resuscitation performance [5]. However, these teams consist solely of healthcare professionals who have shared knowledge and a mutual understanding of which roles should be present and what actions should be expected of the people filling those roles. In groups of immediate responders, it is likely that the people in the group do not know each other, do not know which roles could or should be filled and, consequently, do not know what level of knowledge and expertise to expect from each other. Therefore, the coordination demands are likely to be different compared to intra-hospital teams of healthcare professionals.

Castelao et al. [5] suggest that further research is needed regarding the temporal dynamics of coordination. Engaging with temporal dynamics necessitates analytic methods that allow for the modelling and analysis of temporal structures of group behaviors. Creating a recurrence plot of the timeline of the group’s behaviors enables the calculation of measures of the dynamical structure of the timelines [6, 7]. Commonly used measures in the analysis of the dynamical structures of group behaviors through recurrence plots include recurrence rate of group behaviors, determinism, and Shannon information entropy [8]. These three measures can be used to operationalize stability, predictability, and complexity, respectively [8].

There have been some studies on the temporal dynamics of coordination that have used measures of recurrence rate, determinism, and entropy. Grimm et al. [9] found entropy of communication is negatively related to team performance, and determinism is positively related to team performance in Critical Care Air Transport teams. Gorman et al. [10] found that predictability of communication patterns (i.e., communication determinism) between humans and unmanned aerial systems working together in teams increase as shared experience increase. Stachowski et al. [11] investigated the effect of complexity in interaction patterns in nuclear plant crews responding to a simulated crisis. They found that higher-performing teams used less complex interaction patterns. In summary, these studies indicate that high team performance can be predicted by team interactions with lower complexity (i.e., lower entropy) and higher predictability (i.e., higher determinism).

Although the previous research provides important insight into how dynamical structure in group behavior relates to team performance, there is a lack of more naturalistic investigations of emergent ad-hoc teams. The unmanned aerial systems teams in Gorman et al. [10] collaborated in a digital environment that limited the possible coordinative actions. Stachowski et al. [11] used a physical simulation environment, but the nuclear plant crews consisted entirely of professionals conducting work in their domain of expertise. Therefore, there is a need to explore how the dynamical structure of interaction in ad-hoc groups with mixed levels of relevant expertise affects performance in teams collaborating on a critical task in a physical environment.

Engaging with research on the temporal dynamics of group behavior also necessitates a change in conceptualization from defining a group as a collection of individuals to defining a group as a complex dynamic system [12, 13]. Arrow et al. [12] explain that considering the change of group behavior over time makes linear positivist and reductionist approaches problematic. Conceptualizing groups as complex systems embraces the nonlinearity, recursiveness, and systems aspects of groups that are otherwise missed. For example, the coordinative dynamics of group participants’ movements, heart rate, and electrodermal activity have been shown to predict team coordination states such as implicit and explicit coordination of actions and information in intra-hospital healthcare teams training in a simulation [14]. However, the authors are not aware of any research linking the dynamics of team coordinative states with team performance.

### Aim and purpose

Based on the previous research on coordination in emergency response scenarios, the current exploratory study examines how specific coordination behaviors influence first aid performance. The current study aims to explore how recurrence quantification analysis (specifically, recurrence rate) contributes to the investigation of how the structure of coordinative behaviors in immediate responder groups relates to the groups’ performance in a first aid scenario. This analysis is then compared to aggregated data analysis to identify its relative strengths and weaknesses.

## Methods

Sixteen controlled experimental simulations of a traffic accident were conducted between the 20th of September and 30th of November 2023, with participants in groups of three, at the Center for Disaster Medicine and Traumatology in Linköping, Sweden. The experiment used a mixed two-level design with rater-coded coordinative behaviors as a within-group factor and team composition as a between-group factor (low competence groups vs. mixed competence groups). The current analysis used only the within-group factor, as the purpose was to analyze coordinative behaviors regardless of team competence. However, for transparency, the study sample is described including the between-group competence variable. Results that include the emergency response competence variable are reported elsewhere (see [15, 16]).

Groups that were categorized as having low emergency response competence consisted of three participants with no more than basic first aid education, such as the basic CPR (cardiopulmonary resuscitation) training provided as part of the standard curriculum for Swedish upper secondary schools [17] or through organizations such as the Red Cross. These participants are termed laypeople. Groups with mixed emergency response competence consisted of two laypeople and one healthcare professional with professional experience in emergency response or healthcare, or with training in advanced first aid or other prehospital care qualifications (e.g. Advanced Life Support or Tactical Combat Casualty Care).

**Table 1:** Participants’ demographic characteristics

	Low competence groups		Mixed competence groups		Full sample	
	*n*	%	*n*	%	*n*	%
Groups	10	63	6	37	16	100
Participants	30	63	18	37	48	100
Gender						
Female	15	50	4	22	19	40
Male	15	50	13	72	28	58
Prefer not to answer	0	0	1	6	1	2
First aid education						
Yes	14	47	13	72	27	56
No	16	53	5	28	21	44

### Participants

48 people were recruited from a large university and the surrounding city in southern Sweden using convenience sampling. The recruitment consisted of placing posters on campus and in the city center, sending posters to emergency response stations in the region (to reach emergency response professionals), and posting information about the study on social media using the account of the center for disaster medicine and traumatology (see Tables 1 and 2 for participant demographics). The poster informed potential participants of a monetary reward of 300 SEK (ca. 25 EUR) per hour for participating. First aid groups form ad-hoc [1–3], so special care was taken to maintain the ad-hoc nature of the experimental groups, i.e., the participants did not know in advance that they would act together. Participants were recruited and handled separately before the experiment, and the healthcare professionals were asked to avoid clothing with logos overtly communicating their profession, which could, for example, affect trust [18]. The information that the participants would act together in the same simulation was withheld beforehand as a benevolent deception [19]. Although this does not necessarily support learning, it makes ad-hoc group formation possible. Consent with complete information was re-obtained after the completion of the simulation. No participants chose to withdraw their consent. Ethical approval for the study was obtained from the Swedish Ethical Review Authority before the start of data collection [2023-01980-01].

**Table 2:** Participant years since first aid education, and age demographics

	Low competence groups			Mixed competence groups			Full sample		
	*n*	Mean	SD	*n*	Mean	SD	*n*	Mean	SD
Years since first aid education	11	4.0	3.1	12	6.3	9.9	23	5.2	7.4
Age (years)	30	25.1	7.7	18	31	12.8	48	27.5	10.7

Note. Four participants who received first aid training did not disclose the number of years since their education

### Procedure

**Fig. 1:**
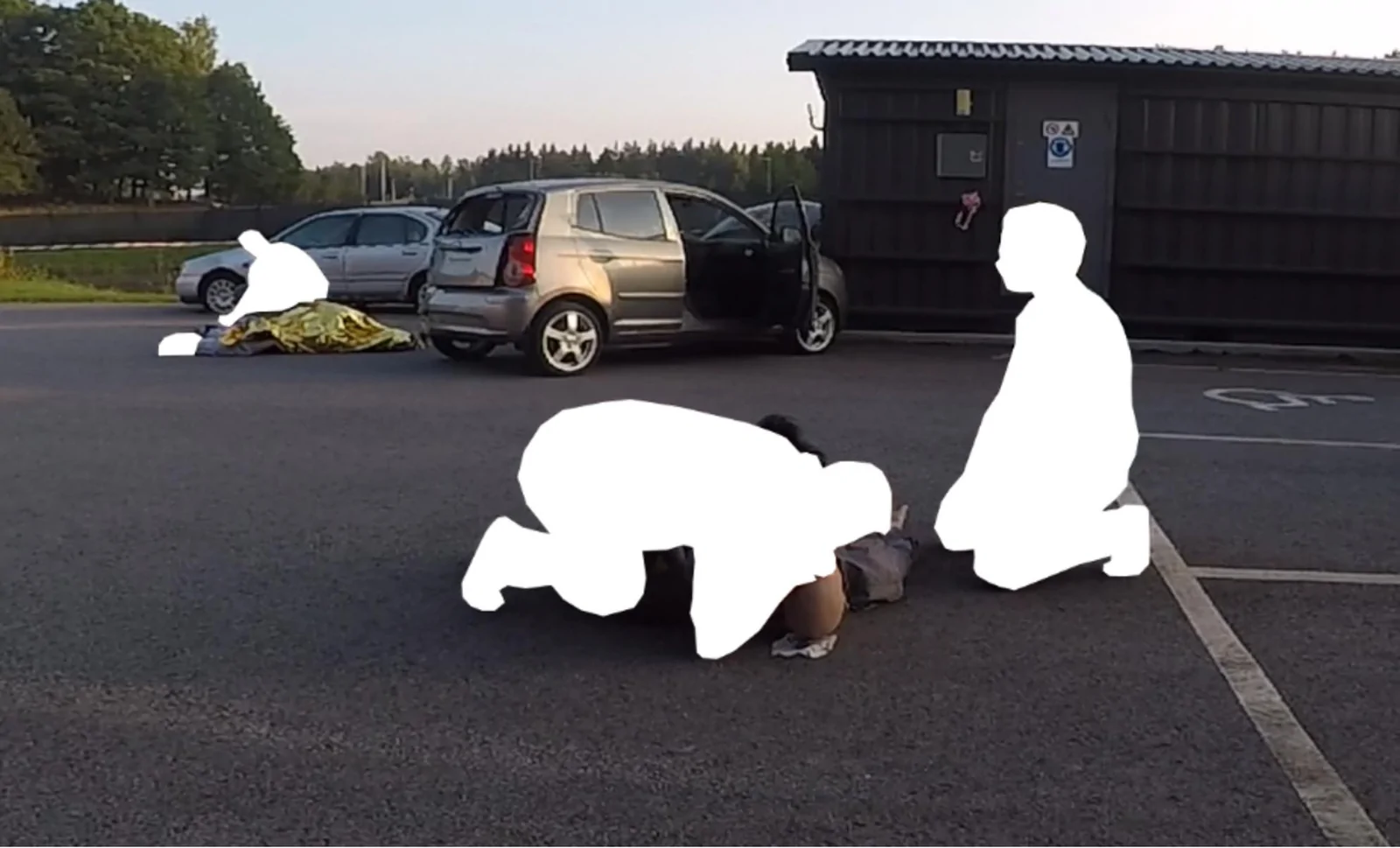
Simulated traffic accident after approximately 5 min. Manikin receiving rescue breaths in the foreground, and a simulated patient with a massive hemorrhage under a rescue blanket in the background

When participants arrived at the simulation site, they were seated separately in the same room in front of a large set of double doors, which separated the pre-simulation area from the simulation area. Participants were separated with mobile room dividers that blocked the line of sight between them. Each participant was addressed individually and privately with information about the study and informed consent. When they were not being addressed, participants wore hearing protection to minimize overhearing the other participants’ instructions and questions, as hearing the other participants receiving the same instructions would leak the information that participants would be acting together in the same scenario. Informed consent to participate was then collected, and each participant was fitted with a small chest-mounted video camera and a lavalier microphone to enable post-experiment performance ratings. They then received instructions for the simulation and, to support their suspension of disbelief [20], they were asked to act as they would if the scenario was real. When the experiment started, all three participants exited the room simultaneously and entered an outdoor fenced-off area where a simulated traffic accident had been staged (see Fig. 1).

The simulated traffic accident was designed with psychological and task fidelity in mind rather than physical fidelity [21]. With regards to the level of technical simulation, it can be considered a level 3 as psychomotor, cognitive, and interpersonal skills were included in the simulation, but the patient models were trained actors or basic manikins and not computerized patient simulators [22]. However, a conflict was present in the suitable level of technical and psychological fidelity as participants consisted of both laypeople and medical professionals which differ in fidelity requirement [23].

The area of the simulated traffic accident contained a car with smoke coming out of the engine bay and two injured people inside; one actor (simulated patient) with a simulated hemorrhage on their upper left arm (TrueClot Tourniquet Application Trainer [24]) and low responsiveness (level two or three on the Reaction Scale [25]), and the second a rescue manikin without pulse or breathing (Rescue manikin PRO [26]). The manikin was used to simulate a victim in cardiac arrest. When participants investigated its physical state, a facilitator provided relevant feedback [27]. The participants were then free to act within this scenario (i.e. experimental simulation [28] where the facilitator acts as an observer rather than a coach or shaping the activities in the simulation [27]) to provide aid to these victims. The simulation was ended after a maximum of 15 min. The scenario was filmed through three additional cameras, of which one was a 360-degree field of view camera. After the scenario, the participants answered a survey regarding their perception of leadership, coordination, authority differentiation, and trust in the scenario. The results from this survey are reported elsewhere [15, 16] and are not used in the current analysis. Complete information about the study was then provided to the participants to clarify the intent of studying teamwork, since the participants had not previously been informed that they would be participating with others in the simulation. Informed consent was then re-obtained. Afterwards, a group interview regarding the participants’ experience of leadership, teamwork, and trust in the scenario was conducted for 30–50 min (not reported in the current article). Finally, there was a debriefing in which the participants could reflect on their experience in the simulation and ask the research leader questions. The total duration of the experiment was about two hours. The participants were then thanked for their participation and the experiment ended.

### Analysis

The first aid performance measure was designed as an expert rated measurement [29] since there are fundamental issues with automated performance measurement in complex simulations [30]. The purpose of the measure was to quantify performance, so it was decided that only taskwork actions would be included in the measure. This excluded teamwork actions, that is, actions that enhance team effectiveness. The performance measure was developed through a workshop with two emergency response professionals and one researcher (author of the article). The description of the scenario was used to specify which types of actions could be assumed to increase the chance of survival of the injured people while not putting immediate responders in unnecessary danger in the real-world equivalent of the scenario. Four categories of behaviors were included: scene safety, hemorrhage control, cardiopulmonary resuscitation, and participant communication with a conscious victim (see Table 3 for evaluation criteria and descriptions of performance categories).

Scene safety aimed to capture how much danger the participants would put themselves in by their actions. Hemorrhage control captured the success of the participants’ attempts to stop the bleeding of the simulated patient using a tourniquet or direct pressure. Cardiopulmonary resuscitation included how quickly the participants started chest compressions and rescue breaths, correctness of depth and compression rate, and if they managed to sustain compressions without pausing. Participants’ communication with the conscious victim included how well the participants kept the simulated patient informed about what they were doing, if they collected information regarding the simulated patient’s medical condition, and if they provided them with psychological support. Three subject matter experts (two of whom are authors of this article) then rated each of the groups independently on Likert-scales from 1 to 7 in the four categories specified in Table 3 by viewing the recordings of the scenarios. This resulted in a total rating score from 4 to 28. The interrater reliability of the average of each rater’s total rating score for each group was calculated through an intraclass correlation coefficient (ICC) estimate with 95% confidence intervals using the R-package irr [31]. The estimate was based on a mean-rating (k = 3) consistency and a 2-way mixed effects model. The ICC showed good reliability, ICC(C,3) = 0.85, 95% CI: 0.65–0.94. Thus, the average of the ratings across the four categories was used as the first aid performance measure in the analysis.

The coordinative behaviors of the participants were coded into a timeline for each group by one of the authors. The timelines were based upon compiled videos for each group featuring all recorded viewpoints except the 360-degree camera. The program DOTE [32] was used to simultaneously view the video and the waveform of the recorded audio when transcribing the timelines to determine when a coordinative act started and ended during behavioral coding. A resolution of 0.1s was used for all timelines due to DOTE displaying time at this resolution. The video was coded according to the coordinative function a given act had in the interaction. Acts that featured two coordinative functions (e.g., a response coupled with a counterquestion) were split and treated as two acts. Any ambiguous codes were discussed with another author. The codes were based on the Co-ACT framework [4], which divides coordinative behaviors into four categories in two dimensions. The first dimension categorizes the coordination behavior as either coordinating an action in the physical world, e.g., speaking up when something appears wrong, or coordinating information, e.g., requesting or providing information. The second dimension concerns the explicitness or implicitness of the behavior. An explicit coordinative behavior is unambiguously coordinating information or actions in pursuit of a shared goal, while implicit coordinative behavior is done in anticipation of need in coordinating information or actions within the group.

**Table 3:** Performance evaluation categories with corresponding evaluation criteria, and descriptions of performance measures by categories

Performance evaluation categories	Evaluation criteria
Scene safety	Considers potential blood infection contamination. Minimizes personal safety risks. Adheres to road safety protocol
Hemorrhage control	See, evaluate & act. Uses direct pressure, tourniquet, or improvised tourniquet. Provides and maintains sufficient pressure on the wound. Time-effective aid.
Cardiopulmonary resuscitation	Initiates as soon as possible. CPR quality (deep compressions, good frequency, correct hand placement, and rescue breaths). No pauses in compressions. Maintains task focus.
Communication with conscious victim	Provides appropriate psychological first aid. Collects relevant medical information. Informs victim of first aid measures performed on them.

Note. The range for all categories is 1–7 (worst to best performance) and the performance rating used in the analysis ranges from 4–28

The dynamic structure of the coordinative behaviors was calculated through a multidimensional recurrence quantification analysis (MdRQA) [33, 34] of each timeline of coded coordinative behavior. In the current analysis, recurrence rate, determinism, and Shannon information entropy [35] were used to measure the dynamical structure of the coordinative behaviors, as they provide measures for the stability, predictability, and complexity of the coordinative behaviors, respectively. In the context of coordination in first aid, recurrence rate (i.e., stability) would be the tendency of the same person in the group to do the same coordinative behaviors again and again or over a longer period of time. This could, for example, be a person acting as a leader who explicitly coordinates the information and actions of others to make sure that the group is focused on the right goal at the right time. Determinism (i.e., predictability) would be the tendency of the group to switch between coordination behaviors in the same order, creating chains of different coordinative behaviors. Groups with high determinism could, for example, be using an iterative procedure of performing actions and then informing others about the results of their actions to enable quick reorganization in response to unexpected changes. Finally, entropy (i.e., complexity) in this study refers to how many different recurring chains of coordinative behaviors occur in the group. This represents the groups’ use of different patterns of coordination during the first aid scenario, as the coordination needed to provide good first aid in the initial moments of the scenario may be different from what is required later.

For the measures of the dynamical structures of different timelines to be comparable, the timelines need to consist of an equal number of data points, and each data point needs to represent the same amount of time. Therefore, the timelines were upsampled so each data point represented 0.1s, and all timelines were shortened to the length of the shortest timeline. This resulted in all timelines representing the coordinative behaviors of the first 10 min 28.8s for their respective groups. The timelines were then split up by participant to enable co-occurring behaviors, and an MdRQA was calculated using the crqa package [34] with each participant’s timelines included as its own dimension. The auto-recurrence plot was calculated with zero delay, one embedding dimension, a radius of 0.1, a minimum diagonal or vertical line of two, Euclidean distance metric, and no inclusion of the line of incidence. The crqa package was unable to calculate a recurrence plot for two low-competence groups due to a lack of sufficiently long time periods of recurring states, and they were thus excluded from the analyses.

A regression between the recurrence rate of the timelines of coordinative behaviors and first aid performance was conducted to analyze how the structure of group dynamics related to first aid performance. Both regression models were checked for extreme outliers and homoscedasticity, and normality of residuals. Normality of residuals met the assumptions for regression for both models. However, due to the low number of data points, the homoscedasticity of residuals did not meet the assumptions of regression, and two potential extreme outliers were found in both models. The significance threshold was set to 0.05.

## Results

**Fig. 2:**
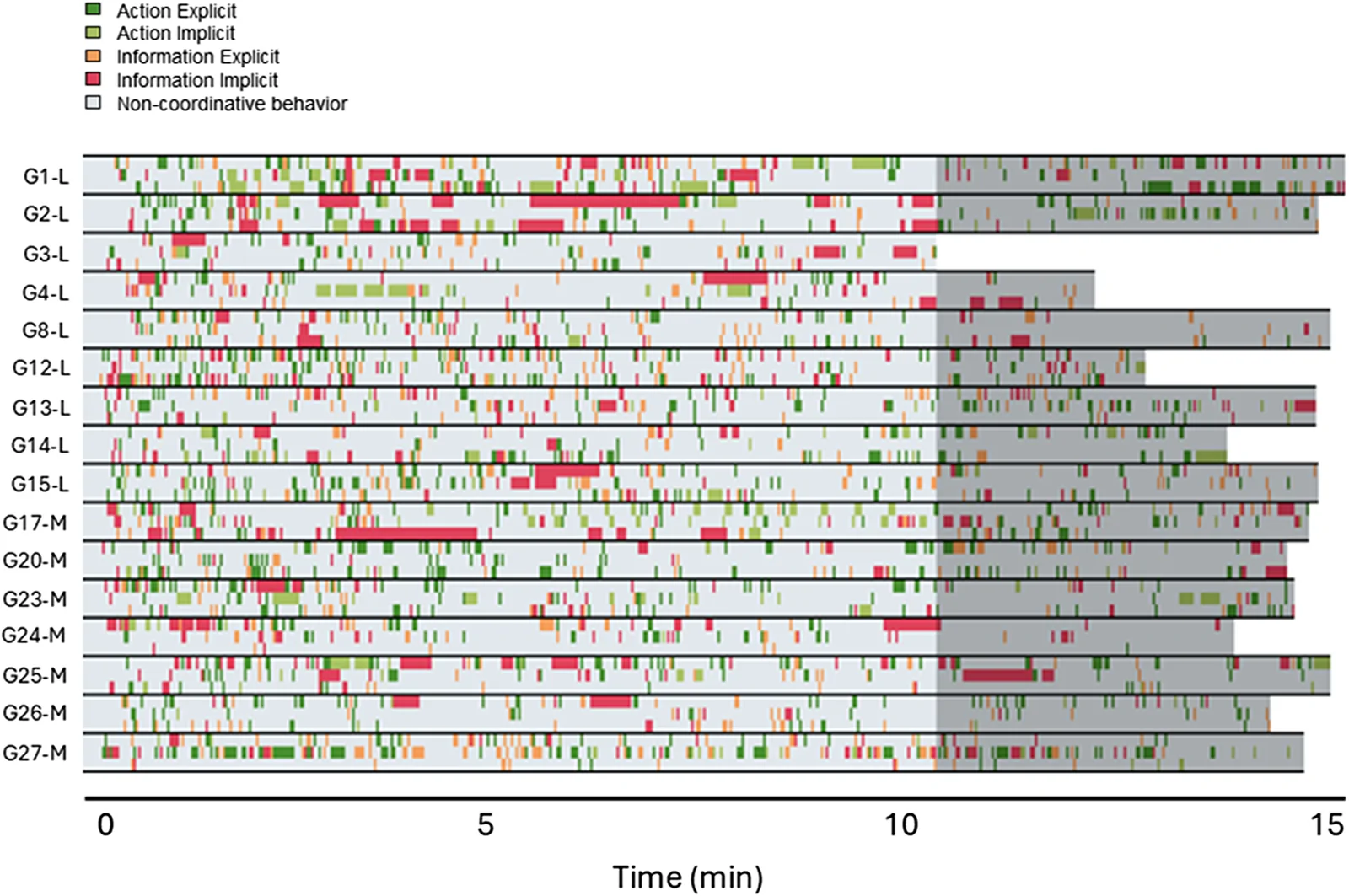
Timelines of coded behaviors for each participant, divided by teams. The shaded area is excluded from the current analysis. Groups with low and mixed emergency response competence are marked with L and M, respectively. Each group contains three separate timelines, one for each participant. G3-L and G14-L were excluded from the analysis due to insufficient time in recurring states

The coding of each participant’s coordinative behaviors resulted in 48 separate timelines showing the points in time at which they implicitly or explicitly coordinated actions or information (see Fig. 2). Two analytical approaches were used: aggregated data analysis and nonlinear data analysis.

### Aggregated data analysis

Coordinative behaviors were aggregated across the 14 groups with complete data to explore their effect on first aid performance (see Table 4), and a multiple regression was performed to investigate how well the coordinative behaviors predicted the performance. Neither the model nor any individual category of coordinative behavior significantly predicted first aid performance, *F*(4,9) = 0.24, *p* = .9, adjusted R^2^ = -0.3 (see Table 5).

Aggregating data removes the temporality of the timelines, which could contain critical information for the prediction of the first aid performance. For example, aggregating makes it impossible to distinguish between groups that often switched between coordinative behaviors and groups that stayed in the same type of coordinative behavior for a longer period before changing. Analyzing temporal variability requires methods without aggregation of data, making it possible to calculate measures of dynamics.

**Table 4:** Descriptions of recurrence rate, first aid performance, and coded coordinative behaviors aggregated across time

	Mean	SD
Recurrence rate	0.006%	0.013
Determinism	91.51%	9.71
Shannon entropy	2.24 *S*	1.08
First aid performance	16.29 score	3.36
Coded behaviors per group		
Action Implicit	17.9s	19.96
Action Explicit	47.92s	20.42
Information Implicit	60.14s	47.91
Information Explicit	22.19s	11.22

Note. 14 groups are included in the analysis. Recurrence rate is the percentage of recurring points in the timeline. Determinism is the percentage of recurring points on a diagonal line. *S* - Shannon entropy is the probability of a recurring point occurring on a diagonal line in a recurrence plot. First aid performance is the sum of four dimensions rated on a scale from 1–7 with a potential range of 4–28. Coded behaviors per group is the time in seconds the groups spent in a coordinative behavior

### Nonlinear data analysis

The dynamical structure of the timelines was analyzed by creating a multidimensional recurrence plot for each group and then calculating multiple measures of the dynamic structure of the coordinative behaviors through a recurrence quantification analysis. Recurrence rate, determinism, and entropy were considered for further analysis. The lowest possible recurrence rate was 0.00016% in comparison to the mean recurrence rate of 0.006%, meaning that the recurrence rate of the groups with incalculable RQAs would be below 0.00016%. The mean and standard deviation of the remaining 14 groups’ recurrence rate, determinism, and Shannon entropy are shown in Table 4.

**Table 5:** Regression of coded coordinated behaviors, aggregated across time, on first aid performance

	b	SE b	β	*p*
Constant	18.3	4.73		< 0.01
Action Implicit	<-0.01	< 0.01	-0.31	0.37
Action Explicit	< 0.01	< 0.01	< 0.01	0.97
Information Implicit	< 0.01	< 0.01	<-0.01	0.99
Information Explicit	<-0.01	0.01	-0.14	0.68

Note. 14 groups included in the analysis. *b* is the unstandardized beta and SE *b* is the standardized error of *b. β* is the standardized beta, and *p* is the probability value

A regression was calculated to investigate how the recurrence rate as a measure of dynamics in the coordinative behaviors of the groups relates to the groups’ first aid performance, *F*(1,14) = 6.6, *p* = .02, adjusted R^2^ = 0.27. Recurrence rate of coordinative behavior was found to be a significant predictor of first aid performance (see Table 6). The standardized beta is negative, which indicates that less recurrence of coordinative behavior increases first aid performance.

**Table 6:** The effect of the entropy of coordinative behavior on first aid performance

	b	SE b	β	*p*
Constant	17.19	0.75		< 0.01
Recurrence rate	-135.26	52.67	-0.57	0.02

Note. 14 groups are included in the analysis. *b* is the unstandardized beta and SE *b* is the standardized error of *b. β* is the standardized beta, and *p* is the probability value

## Discussion

The duration of any coordinative behavior, as categorized according to Co-ACT, was not found to predict first aid performance (see Table 5). Coordination has been found to improve intra-hospital CPR, in particular clear and proactive communication [5]. Thus, the results from the current out-of-hospital exploratory study do not correspond to previous studies in the intra-hospital domain. One possible explanation for the difference could be that providing first aid (i.e., completing task work) is possible without the need to coordinate in cases where there are more immediate responders than injured people.

In the current exploration of measures of dynamical structures’ relation to team performance, recurrence rate as a measure of stability in coordinative behaviors in immediate responder groups was found to negatively predict first aid team performance (see Table 6). The current exploratory study extends the findings to group interactions, both in communication (information coordination in Co-ACT) and coordinative actions, and to ad-hoc groups doing taskwork where all or most group members are in an unfamiliar and critical situation. In emergency response, the response can be thought of as an effort to bring order to a chaotic situation while maintaining sufficient flexibility to meet the demands of the emergency [36]. Decreasing stability could therefore be expected to increase the flexibility of the response. Ashby’s law of requisite variety posits a similar claim, i.e. the controller of a complex system must have at least the same possible variability in their controls as there is variability in the complex system they are trying to control [37]. Requisite variety has been used to explain the positive impact of increased individual variability on system performance [38, 39] and more recently to explain team adaption [40]. A decrease in stability in coordinative behaviors allows immediate responders to use other behaviors that are not measured with Co-ACT, such as the taskwork of first aid. The non-significant predictiveness of aggregated coordinative behaviors and the negative effect of coordination stability suggest that first aid performance in the current exploratory study did not require coordination behaviors. Simply put, the results suggest that ad-hoc immediate responder groups should be allowed flexibility in their response in scenarios with multiple injured people.

Meaningful comparison between first aid groups’ dynamical structures is only possible through a controlled experimental simulation. Previous research on teamwork has put forward the need to develop and use methods for analyzing the dynamics of teamwork (see for example [12, 41]), . The disaster response subject area could make much use of methods to measure dynamics, as facing complex scenarios is a central part of the responsibility of disaster and emergency response professionals [36]. In the current exploratory study, the first aid response by immediate responders is modeled as a complex adaptive system where the performance of first aid depends on interaction and coordination over time by the immediate responders. While limiting the model of the response to solely coordinative behavior limits the ability to identify interdependence between the responders, it also allows for investigation of interaction structures that are not available through research paradigms that involve data aggregation.

Disaster and emergency responses are sometimes described as chaotic by researchers and practitioners [36]. It makes intuitive sense to describe a phenomenon that is difficult to predict and control as chaotic, but it also has ontological implications in that chaos is a state in a complex adaptive system with nonlinear dynamical changes in the system states. Additionally, disaster response data has been shown to exhibit nonlinear characteristics [42]. Using complexity methods such as multidimensional recurrence quantification analysis makes it possible to study the changes in such a system. Therefore, using controlled scenarios with teams in complex adaptive systems provides an environment to study disaster and emergency response as a chaotic phenomenon while maintaining experimental control. From this, we are able to draw conclusions and make generalizations about important coordinative processes that would otherwise be difficult to study in real situations.

### Limitations

The current exploratory study provides a unique exploration into the dynamics of cooperation in ad-hoc immediate responder groups. However, it is an exploratory study and there are notable limitations with regard to the data and analysis. The sample size of the study is small, and the results from the regressions should thus be interpreted cautiously as they are at risk for overfitting. The analysis was limited to only one measure, recurrence rate, for this reason. Future studies should, if possible, include other measures such as determinism or entropy. Similarly, the behavioral coding was done by one coder and therefore analyzing the reliability of the coding is impossible. This limits the reliability of the results that are based on the coded data and future research should include multiple coders and check coding reliability (See for e.g. [43]). All timelines were shortened to the same length as the shortest timeline to enable comparable MdRQA measures (see shaded area of timelines in Fig. 2 for data not included). Therefore, only the first 10 min 28s of the scenario were included in the analysis, and any potential aspects of the coordinative behavior crucial to the performance score in the last 5 min of the scenario were left out. This limits the analysis to the first two thirds of the simulations. Although early intervention is the most important part of effective first aid, the later aspects of the coordination, though less important, should be captured. Therefore, future work should strive to minimize the difference between the shortest and longest scenario times to avoid removing collected data from the analysis.

The timelines for two groups (group G3-L and G14-L) had insufficient time of recurring states, resulting in no data from the MdRQA for these groups, and thus they were not included in the nonlinear analysis. The main effect found in the nonlinear analysis was that recurrence rate was negatively predictive of first aid performance. The two excluded groups’ first aid performance scores were both less than the sample mean of 16.4 (G3-L 15.67, G14-L 14.33). The insufficient recurring states of coordination behavior means that the inclusion of the two groups could have weakened the negative effect of recurrence rate on first aid performance that was found in the nonlinear data analysis.

The MdRQA enables calculation of similarities in the states that a group is in throughout the scenario, but it is limited to the exact recurrence of the state. This means that a state with one participant enacting explicit information coordination will only be counted as recurring if the same participant engages in the same coordinative behavior. If a different participant also explicitly coordinates information, it will not be counted as recurring with the previously mentioned state, even though the two states might be functionally identical to the group. This is likely part of the reason for two groups having noncomputable recurrence plots. Four coding dimensions for three participants could thus be the upper limits of when MdRQA is useful as an analysis method in a teamwork context. It could also be the case that the use of zero time delay and one embedding dimension, while recommended for unordered categorical data [34], is not optimal for MdRQA. MdRQA is recommended over other recurrence quantification analysis variants when analyzing team-level collaboration phenomena as it avoids aggregation of data [44]. However, alternative complexity methods may be more useful, e.g. state-space smoothing [45], especially when analyzing groups with more participants and coding schemas with more dimensions than the current study.

The Co-ACT framework was developed primarily for use in organizing and measuring teamwork of acute care teams [4]. These teams feature members with specialized roles and competency in treatment. The groups featured in this study had either low or mixed competency levels, which makes it possible that the underlying assumptions that resulted in the codes of the framework are not ideally suited for these kinds of groups. The groups showed several instances of tentative behaviors and were often giving and receiving low-level feedback to each other. While the Co-ACT framework can represent these behaviors, it also aggregates many different kinds of behaviors into one code. It is possible that a larger number of codes could lead to better distinction and possibly more insightful analysis in the case of low or mixed competency teams.

Studying human behavior in simulated scenarios may limit the ecological validity [28]. However, gaining reliable access to the phase of emergency and disaster response that precedes the arrival of professional responders is impossible without simulation. As such, the tradeoff between collecting meaningful data on how cooperation changes over time and ecological validity is necessary to gain access to the subject at all.

Participants were informed that they would receive a monetary reward for participation, which could influence people’s desire to participate even though they would not otherwise have participated. This potential skewing of the recruited participants together with the convenience sampling limits the generalizability of the results.

Future work should aim to investigate ad-hoc immediate responder groups using other teamwork constructs (e.g., team adaptation) and complexity methods (e.g., moving window analysis) [40]. Team adaptation could be useful to understand if there are different behaviors in response to the first aid scenario as it unfolds. In the current exploratory study, the relation of stability and performance could be different at different times during the provision of first aid as the work continues. Such analyses could be better at making predictions regarding which specific coordination behavior is better at which time in a first aid response. Therefore, continuing the current work could lead to more practical applications.

## Conclusion

In the current exploratory study, the recurrence rate was used as a measure of the stability in the dynamical structure in first aid groups’ coordinative behavior in a controlled simulated first aid scenario. The recurrence rate of coordinative behaviors was found to be negatively related to first aid team performance as rated by subject matter experts. Groups that showed recurrence of coordinating information and action, either explicitly or implicitly, provided a first aid response that was quicker and of higher quality with regards to hemorrhage control, CPR, communication with injured people, and maintaining a safe environment. Additionally, aggregated measures of coordinative behaviors did not predict first aid performance. This indicates that measures of temporal dynamics are needed to understand how cooperation in immediate responder groups drives first aid performance, and the need to use controlled scenarios to study how dynamical complexity affects performance in disaster and emergency response scenarios.

## Data Availability

The datasets used and analyzed during the current study are available from the corresponding author on reasonable request but restrictions apply to the availability of these data due to some data containing non-anonymized information.

## Abbreviations

MdRQA: Multidimensional recurrence quantification analysis
CPR: Cardiopulmonary resuscitation
Co-ACT: Observing Coordinative Behavior in Acute Care Teams
